# Peripheral lymphocyte populations in ovarian cancer patients and correlations with clinicopathological features

**DOI:** 10.1186/s13048-022-00977-3

**Published:** 2022-04-11

**Authors:** Shuang Ye, Wei Chen, Yuwei Zheng, Yutuan Wu, Libing Xiang, Teng Li, Bo Ping, Xiaoming Zhang, Huijuan Yang

**Affiliations:** 1 Department of Gynecologic Oncology, Fudan University Shanghai Cancer Center, Shanghai, China; 2 Department of Oncology, Shanghai Medical College, Fudan University, Shanghai, 200032 China; 3 Department of Obstetrics and Gynecology, Minhang Hospital, Fudan University, the Central Hospital of Minhang District, Shanghai, China; 4 Department of Pathology, Fudan University Shanghai Cancer Center, Shanghai, China; 5 Unit of Innate Defense and Immune Modulation, Key Laboratory of Molecular Virology and Immunology, Institute Pasteur of Shanghai, Chinese Academy of Sciences, Beijing, China

**Keywords:** Ovarian Neoplasms, Lymphocyte, Subpopulation, Flow cytometry

## Abstract

**Background:**

To investigate the alterations of peripheral lymphocyte subpopulations in ovarian cancer patients compared to benign or borderline counterparts. The possible clinicopathological implications were also evaluated.

**Methods:**

We enrolled 112 treatment-naive ovarian cancer patients, 14 borderline tumor patients and 44 benign tumor patients between 09/2016 and 01/2019. Flow cytometry was used to measure the peripheral lymphocyte subsets consisting of T cells (CD3^+^, CD3^+^CD4^+^, CD3^+^CD8^+^ and CD8^+^CD28^+^), regulatory T cells (Tregs, CD4^+^CD25^+^CD127^−^), natural killer cells (NK cells, CD3^−^CD56^+^) and B cells (CD19^+^).

**Results:**

Most ovarian cancer patients were high-grade serous carcinoma (84.8%), followed by clear cell carcinoma (8.03%). Late-stage tumor (FIGO III + IV) accounted for 82.1%. The study showed that the proportions of peripheral lymphocyte subsets underwent apparent changes in ovarian cancer patients. We observed elevated levels of Treg cells in patients with both ovarian borderline and malignant tumor compared to those with benign tumors, which achieved statistic significance. In contrast, CD3^+^CD8^+^ T and CD8^+^CD28^+^ T cells were significantly lower in ovarian cancer patients. Interestingly, low level of B cells was correlated to clear cell carcinoma (*P* = 0.024), advanced tumor (*P* = 0.028) and platinum-resistant recurrence (*P* = 0.014). Regarding the changes of lymphocyte subsets after surgery, CD8^+^CD28^+^ T cells had a significant decreasing tendency (*P* = 0.007) while B cells were the opposite (*P* < 0.001).

**Conclusions:**

Ovarian cancer patients have altered circulating lymphocyte profile (elevated Treg cell, depressed CD3^+^CD8^+^ T and CD8^+^CD28^+^ T cells). Low level of B cells might be related to disease aggressiveness, and it recovered after the removal of tumor, which merits further study.

**Supplementary Information:**

The online version contains supplementary material available at 10.1186/s13048-022-00977-3.

## Highlights

1 Ovarian cancer patients have altered circulating lymphocyte profile

2 Low level of B cells might be related to disease aggressiveness, and it recovered after the removal of tumor.

**Supplementary Information:**

The online version contains supplementary material available at 10.1186/s13048-022-00977-3.

## Background

Ovarian cancer remains the most lethal gynecologic malignancy. The role of the immune response in ovarian cancer is well demonstrated in the literatures [1–3]. Zhang and colleagues first reported a positive association between the number of tumor infiltrating lymphocytes and survival outcome [1]. Other studies arrived at the same conclusion, which was summarized in a meta-analysis [4]. Patients with a more robust immune response (documented by the existence of lymphocytes infiltrating within the tumor) have better response to chemotherapy and survival [5].

On the other hand, the role of peripheral lymphocytes has also been investigated in ovarian cancer, with a focus on regulatory T (Treg) cells [6–8]. In addition to T lymphocytes, B lymphocytes and Natural Killer (NK) cells are also important in maintaining the immunological balance. The alterations of lymphocyte subsets have been studied in several kinds of cancer, including hepatocellular carcinoma [9], head and neck squamous carcinoma [10], pancreatic cancer [11] and lymphoma [12].

In the current study, we aimed to depict the circulating lymphocyte profile by flow cytometry in ovarian cancer patients compared to those with ovarian benign and borderline tumors. Further, the results of flow cytometry were analyzed in relation to clinicopathological features including histology, tumor stage, and platinum response. Finally, we compared the pre- and post-treatment lymphocyte distribution.

## Materials and methods

### Study patients and data collection

**Fig. 1:**
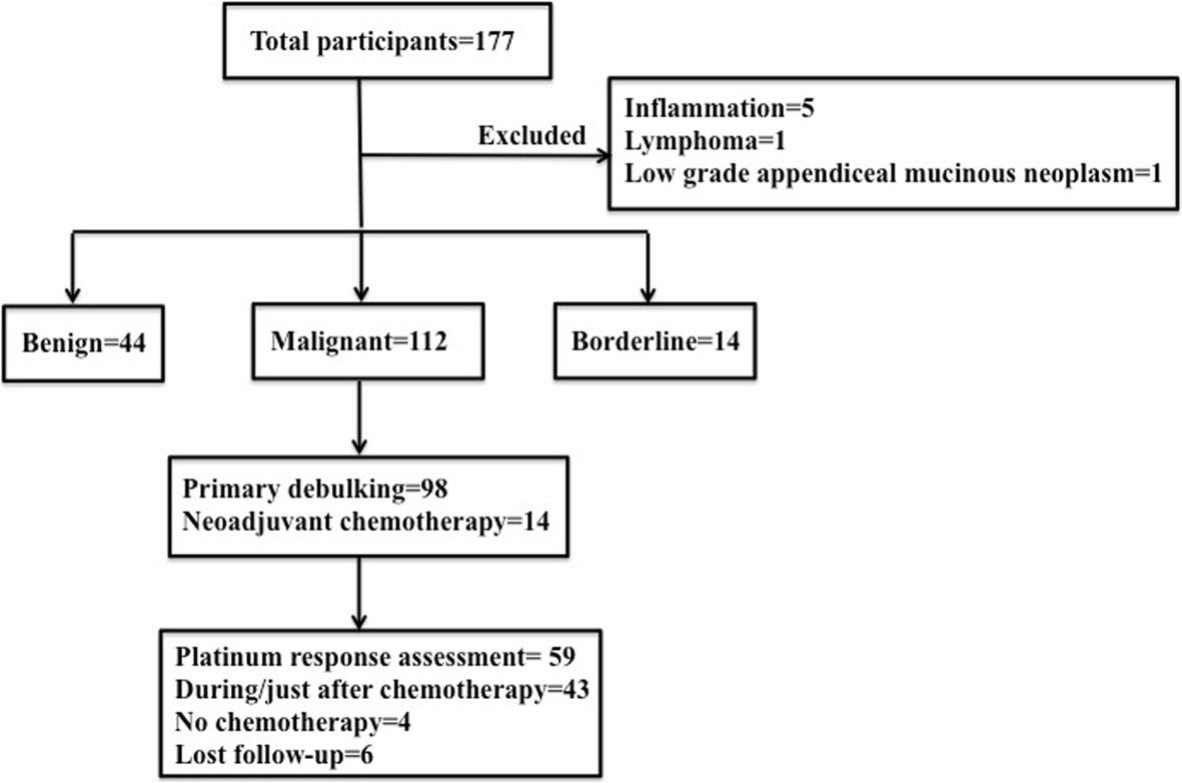
Schematic of patients included in the present study

The study was approved by the ethics committee of Fudan University Shanghai Cancer Center. We included all the patients highly suspicious for ovarian cancer and those with clinical benign adnexal mass from September 2016 to January 2019. The inclusion criteria were listed as follows: 1)A pathology confirmed diagnosis of ovarian cancer, benign ovarian tumor and borderline ovarian tumor; 2) No preoperative treatment including chemotherapy and laparotomy for those suspicious for ovarian cancer; 3) No significant past medical histology such as autoimmune diseases; 4) Without any sign of infection. Informed consent was obtained. Figure 1 presents the flow chart of patients throughout the study.

Clinicopathological features were retrospectively abstracted from the electronic medical record. Data collection included age at first diagnosis, International Federation of Gynecology and Obstetrics (FIGO) stage [13], pre-treatment serum cancer antigen 125 (CA 125), intra-operative findings, adjuvant chemotherapy and follow-up information. Early-stage patients (FIGO I + II) underwent complete staging, and those with advanced tumor (FIGO III + IV) received debulking surgery. Optimal cytoreductive surgery (CRS) was defined as residual disease less than (or including) 1 cm after primary debulking. Patients were considered to have platinum-sensitive disease if the interval time was > 6 months from the completion of the last platinum based chemotherapy to disease recurrence.

### Blood sample collection and flow cytometry

Fasting venous blood samples were collected in EDTA-anticoagulated tubes at admission after informed consent. The time interval from blood collection to surgery/neoadjuvant chemotherapy is usually less than seven days. For ovarian cancer patients, post-operative blood samples were also collected before discharge, usually within 1–2 weeks. The flow cytometry protocol was introduced specifically in a previous publication from our institution [11]. The fresh peripheral blood samples were analyzed within 24 h since collected. The cytometer FACS CANTO II (BD Biosciences, USA) were used for flow cytometry analyzing. Cell surface staining with fluorochrome-conjugated antibodies was performed in the dark at 4℃ for 30 min, then rewashed and re-suspended in PBS twice. The DIVA and CANTO software (BD Bioscience, USA) were used for logic gating and data analyzing. The fluorochrome-conjugated antibodies were shown in Supplementary Table 1, while the logic gating and analyzing figures for flow cytometry were presented in Supplementary Figures.

### Statistical analysis

Statistical Package for Social Science (SPSS) (Version 20.0, SPSS, Inc., Chicago, IL, USA) and GraphPad Prism (Version 6.0, GraphPad Software, Inc., La Jolla, CA, USA) were used for the statistic analyses. Clinicopathological parameters and lymphocyte subsets were presented using descriptive statistics. Medians and ranges were applied for continuous variables, while proportions for categorical one. Comparisons were performed by the parametric Student's T tests and non-parametric Mann–Whitney U tests as appropriate. All *P* values reported were two tailed, and *P* < 0.05 was considered statistically significant.

## Results

### Patient characteristics

A total of 177 participants were involved and seven were excluded after final diagnosis (Fig. 1). The number of malignant, borderline, and benign cases was 112, 14 and 44, respectively. As shown in Table 1, 98 ovarian cancer patients underwent upfront surgery while 14 received neoadjuvant chemotherapy. The median age of ovarian cancer patients was 56 years (range, 36–74). In terms of histologic subtype, high-grade serous carcinoma accounted for the majority (84.8%), followed by clear cell carcinoma (8.03%). Over eighty percent (92/112, 82.1%) patients presented with late-stage tumor (FIGO III + IV). Optimal debulking was achieved in 78.6% patients. Clearly seen from Fig. 1, 59 patients were available for platinum response assessment. Of them, platinum-resistant recurrence represented 23.7%.

### Lymphocyte subsets in peripheral blood: ovarian benign tumor vs. borderline tumor vs. malignant tumor

By flow cytometry, the circulating lymphocyte subpopulations were measured, including T cells (CD3^+^, CD3^+^CD4^+^, CD3^+^CD8^+^ and CD8^+^CD28^+^), regulatory T cells (Tregs, CD4^+^CD25^+^CD127^−^), natural killer cells (NK cells, CD3^−^CD56^+^) and B cells (CD19^+^) (Table 2).

**Table 1:** Clinicopathological information of ovarian cancer patients (*n* = 112)

Variables	
Age (years), median (range)	56 (36–74)
Neoadjuvant chemotherapy (%)	14 (12.5%)
Histology	
High-grade serous carcinoma	95 (84.8%)
Clear cell carcinoma	9 (8.03%)
Endometrioid	2 (1.8%)
Mucinous	2 (1.8%)
Carcinosarcoma	3 (2.7%)
Squamous	1 (0.9%)
FIGO stage (%)	
I	15 (13.4%)
II	5 (4.5%)
III	64 (57.1%)
IV	28 (25.0%)
Pre-treatment serum CA 125 (U/mL), median (range)	746.4 (10–5000)^a^
Residual disease (%)	
≤ 1 cm (optimal)	88 (78.6%)
> 1 cm (suboptimal)	24 (21.4%)
Platinum response (%)^b^	
Sensitive	45 (76.3%)
Resistant	14 (23.7%)

^a^The upper limit of CA 125 detection is 5000

^b^A total of 59 patients were available for platinum response assessment

*Abbreviations*: *FIGO* The International Federation of Gynecology and Obstetrics, *CA125* Cancer Antigen

**Fig. 2:**
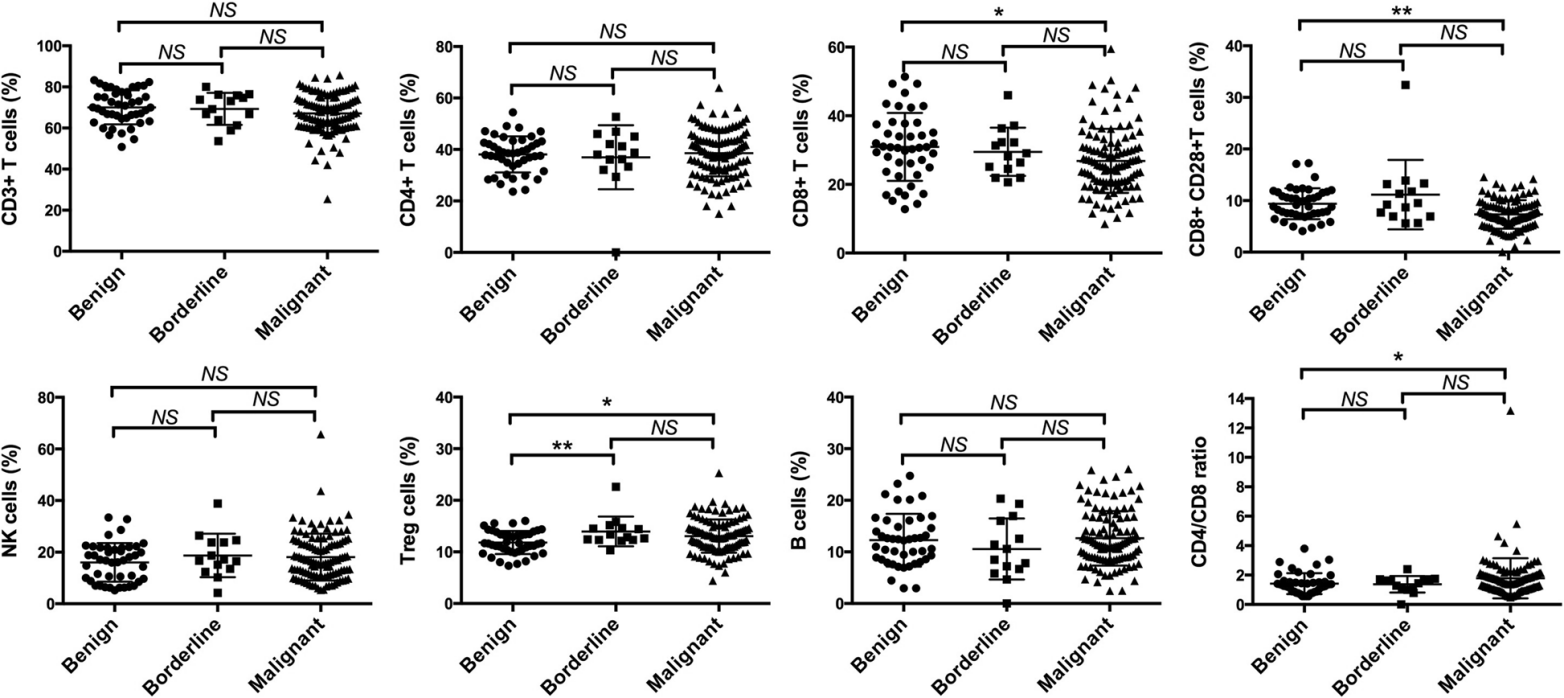
Comparisons of circulating lymphocyte subsets in different populations (Ovarian benign tumor Vs. borderline tumor Vs. malignant tumor). Abbreviations: NS = not significant; *, *P* < 0.05; ** *P* < 0.01

The results showed that the distribution of peripheral lymphocyte subpopulation underwent apparent changes in ovarian cancer patients (Fig. 2, Table 2). CD3^+^CD8^+^ T cells were significantly lower in ovarian cancer patients than benign disease (malignant Vs. benign: 26.84 ± 9.33 Vs. 30.91 ± 9.90, *P* = 0.017). There was also a trend towards lower level of CD8^+^CD28^+^ T cells in ovarian cancer patients compared to benign counterparts (malignant Vs. benign: 7.31 ± 2.77 Vs. 9.39 ± 3.00, *P* < 0.001). No significant difference was observed among three groups concerning CD3^+^ T cells, CD3^+^CD4^+^T cells, NK cells and B cells. Interestingly, increased proportions of Tregs were noted in both malignant and borderline tumor as compared to benign disease (malignant Vs. benign: 13.05 ± 3.22 Vs. 11.82 ± 2.23, *P* = 0.021; borderline Vs. benign: 13.96 ± 2.89 Vs. 11.82 ± 2.23, *P* = 0.005). CD4/CD8 ratio was higher in ovarian cancer patients than benign tumor (malignant Vs. benign: 1.78 ± 1.37 Vs. 1.42 ± 0.71, *P* = 0.038, Mann–Whitney U test).

**Table 2:** Distribution of peripheral lymphocyte subpopulation in different cohort

	Benign(*n* = 44)	Borderline(*n* = 14)	Malignant(*n* = 112)	*P*
CD3^+^ T cells (%)	70.06 ± 8.27	69.33 ± 7.76	67.15 ± 9.49	*P1* = 0.769^*^ *P2* = 0.075^*^ *P3* = 0.825^*^
CD3^+^CD4^+^T cells (%)	38.09 ± 7.00	37.00 ± 12.42	38.59 ± 9.05	*P1* = 0.680^*^ *P2* = 0.738^*^ *P3* = 0.552^*^
CD3^+^CD8^+^ T cells (%)	30.91 ± 9.90	29.50 ± 7.03	26.84 ± 9.33	*P1* = 0.624^*^ *P2* = 0.017^*^ *P3* = 0.307^*^
CD8^+^CD28^+^ T cells (%)	9.39 ± 3.00	11.15 ± 7.74	7.31 ± 2.77	*P1* = 0.176^*^ *P2* < 0.001^*^ *P3* = 0.054^*^
NK cells (CD3^−^CD56^+^) (%)	16.03 ± 7.46	18.72 ± 8.42	18.10 ± 9.06	*P1* = 0.259^*^ *P2* = 0.179^*^ *P3* = 0.810^*^
Tregs (CD4^+^CD25^+^CD127^−^)(%)	11.82 ± 2.23	13.96 ± 2.89	13.05 ± 3.22	*P1* = 0.005^*^ *P2* = 0.021^*^ *P3* = 0.317^*^
B cells (CD19^+^) (%)	12.27 ± 5.13	10.56 ± 5.91	12.64 ± 5.28	*P1* = 0.299^*^ *P2* = 0.689^*^ *P3* = 0.172^*^
CD4/CD8 ratio	1.42 ± 0.71	1.37 ± 0.56	1.78 ± 1.37	*P1* = 0.478^#^ ***P2* = 0.038**^**#**^ *P3* = 0.274^#^

^a^Numbers were presented as mean ± standard deviation

^b^*P* values with statistical significance were denoted. *P*1 = Borderline Vs. Benign;*P*2 = Malignant Vs. Benign; *P*3 = Malignant Vs. Borderline

^*^Paired T test

^#^Mann–Whitney U test

In summary, we observed elevated levels of Treg cells in the circulatory blood of both ovarian borderline and malignant tumor patients. CD3^+^CD8^+^ T and CD8^+^CD28^+^ T cells were significantly lower in ovarian cancer patients.

### Associations of lymphocyte subset and clinicopathological parameters

**Fig. 3:**
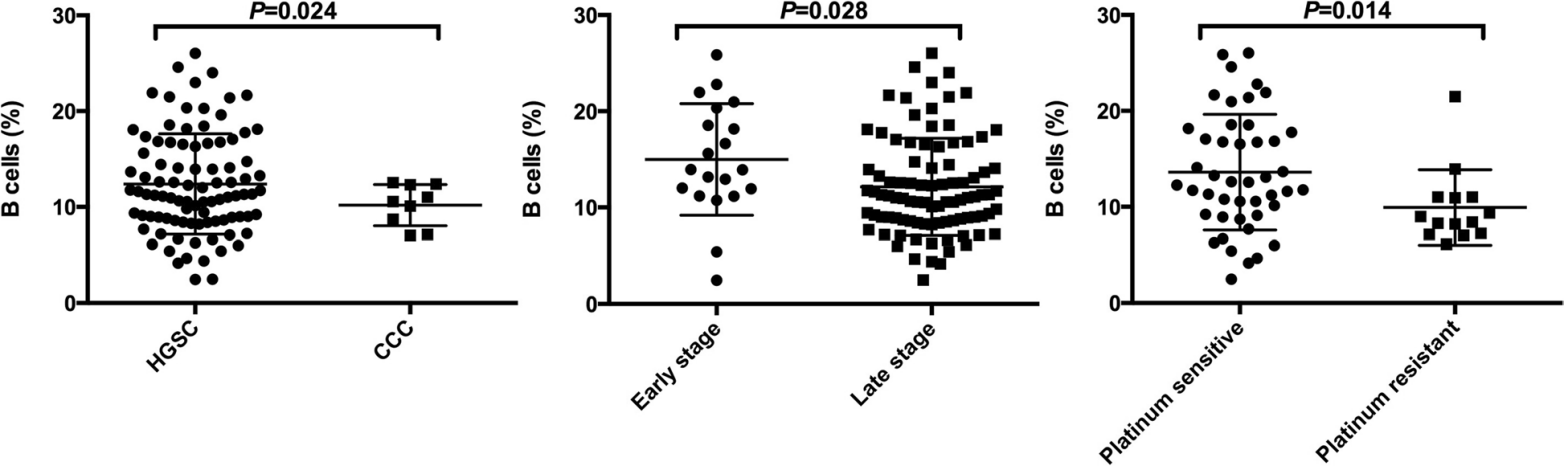
Associations between B cell and clinicopathological features. Abbreviations: HGSC = High-Grade Serous Carcinoma; CCC = Clear Cell Carcinoma

We further investigated the possible associations between lymphocyte subpopulation and clinicopathological parameters, including histology, stage and platinum response. Table 3 depicts that low level of B cells was related to clear cell carcinoma, late stage tumor and platinum-resistant recurrence (Fig. 3). No other correlation was observed.

**Table 3:** Correlations between peripheral lymphocyte subsets and clinicopathological features in ovarian

Variables	Histology			FIGO stage			Platinum Response		
	HGSC	CCC	*P*	Early (I + II)	Late (III + IV)	*P*	Sensitive	Resistant	*P*
CD3^+^ T cells (%)	66.88 ± 9.46	73.27 ± 8.07	0.053	66.56 ± 10.91	67.27 ± 9.21	0.763	66.99 ± 7.44	66.07 ± 15.14	0.835
CD3^+^CD4^+^T cells (%)	38.43 ± 9.22	42.18 ± 7.65	0.241	39.19 ± 8.64	38.47 ± 9.18	0.748	39.48 ± 7.84	36.67 ± 12.59	0.458
CD3^+^CD8^+^ T cells (%)	26.49 ± 8.90	28.82 ± 10.94	0.464	26.73 ± 11.27	26.87 ± 8.93	0.951	27.63 ± 8.61	29.39 ± 12.85	0.565
CD8^+^CD28^+^ T cells (%)	7.07 ± 2.80	8.51 ± 2.88	0.145	7.70 ± 2.52	7.23 ± 2.83	0.489	7.53 ± 2.90	6.67 ± 3.58	0.374
NK cells (CD3^−^CD56^+^) (%)	18.37 ± 8.95	14.30 ± 6.96	0.188	17.89 ± 10.08	18.15 ± 8.88	0.908	17.15 ± 7.34	21.64 ± 15.33	0.323
Tregs (CD4^+^CD25^+^CD127^−^)(%)	12.91 ± 3.22	13.79 ± 4.14	0.445	13.20 ± 3.07	13.02 ± 3.26	0.830	12.50 ± 2.63	12.47 ± 4.24	0.976
B cells (CD19^+^) (%)	12.40 ± 5.24	10.20 ± 2.15	**0.024**	14.99 ± 5.81	12.13 ± 5.05	**0.028**	13.61 ± 6.03	9.86 ± 4.07	**0.014**
CD4/CD8 ratio	1.67 ± 0.85	1.74 ± 0.91	0.818	2.40 ± 2.69	1.64 ± 0.82	0.253	1.88 ± 1.83	1.55 ± 1.00	0.386

*Abbreviations: HGSC* High-Grade Serous Carcinoma, *CCC* Clear Cell Carcinoma, *FIGO* The International Federation of Gynecology and Obstetrics

**Fig. 4:**
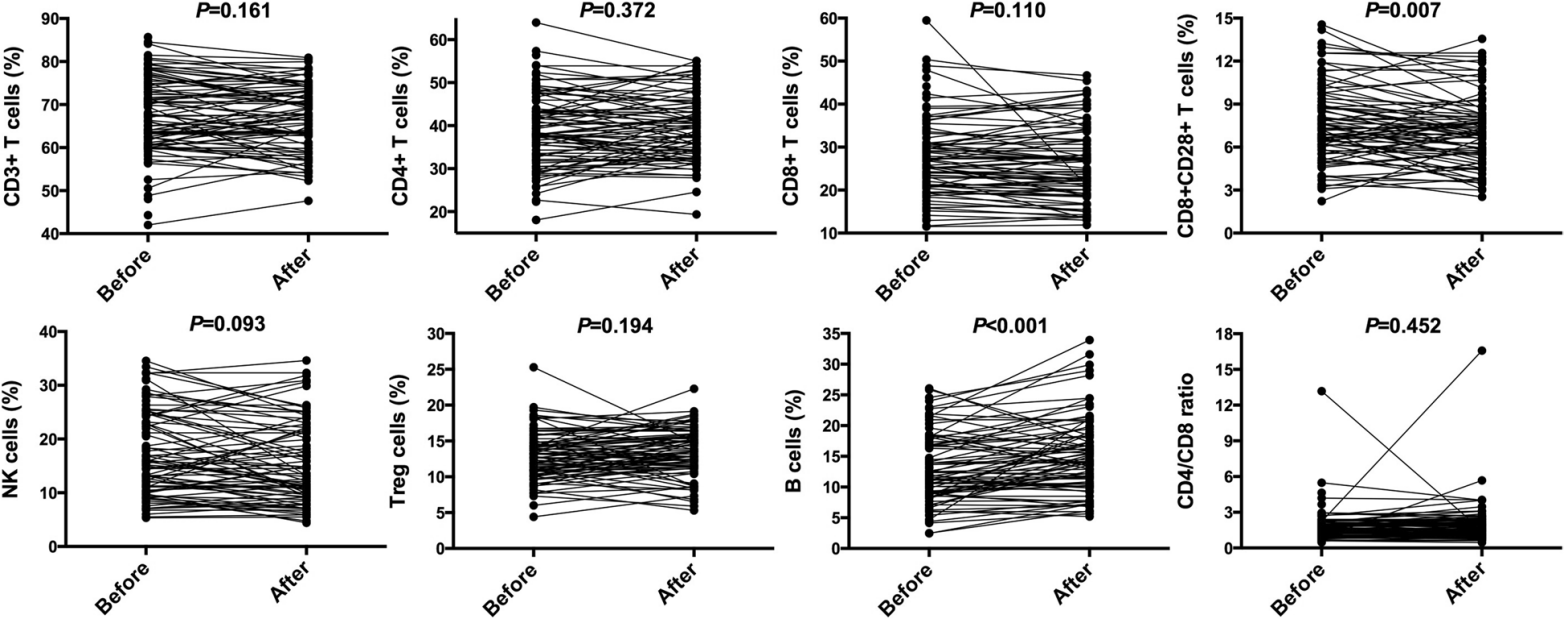
Changes in peripheral lymphocyte distributions in 98 ovarian cancer patients after upfront surgery. Abbreviations: Before = before surgery; After = after surgery

Among the 98 ovarian cancer patients who received upfront surgery, we evaluated the effect of surgery on the distributions of circulating lymphocyte subsets (Fig. 4). The post-operative level of CD8^+^CD28^+^ T cells was lower than the pre-operative one with statistic significance (7.63 ± 2.70 vs. 7.09 ± 2.43, *P* = 0.007). A remarkable increase of B cells was noted after primary debulking surgery (12.92 ± 5.69 vs.14.96 ± 6.28, *P* < 0.001). Other variables remained unchanged before and after operation.

## Discussion

It has been well accepted that ovarian cancer patients present an immunosuppressive status, which was confirmed again by our work. In the present study, we observed the apparent alterations of circulating lymphocyte profile in ovarian cancer patients compared to patients with benign tumors or borderline tumors. Not surprisingly, CD3^+^CD8^+^ T cells and CD8^+^CD28^+^ T cells were depressed in ovarian cancer patients compared to patients with benign tumor. Based on the expression of CD28^+^, CD8^+^ T cells could be divided into CD8^+^CD28^+^ cytotoxic lymphocytes and CD8^+^CD28^−^ inhibitory T cells [14]. Besides, the immunosuppressive Treg cells were significantly higher in patients with ovarian borderline tumor and malignant cancer. Increased circulating Treg cells have been reported in patients with different kinds of malignant tumor, including ovarian cancer [6, 11, 15, 16].

The most interesting finding was that decreased proportions of circulating B cells was correlated to clear cell carcinoma, advanced stage, and platinum resistance. The role of B cells in ovarian cancer is more difficult to discern [17, 18] and remains controversial [18]. Yang and colleagues measured CD19^+^ B cells in omental specimens in 49 high-grade epithelial ovarian cancer patients by immunohistochemistry [18]. The conclusion was that increased B cell infiltration was associated with worse survival [18]. Our study, from the perspective of peripheral blood, illustrated that decreased circulating B cells might be representative of disease aggressiveness (clear cell carcinoma, late stage and platinum insensitivity), which warrants further investigation.

The possible role of peripheral lymphocyte subset in differential diagnosis of ovarian mass leaves to be further studied. In the clinical setting, the approach to the patients with adnexal mass usually includes patient history, serum tumor markers and imaging modality (ultrasound and/or Magnetic Resonance Imaging). There is no universally accepted classification system for defining the risk of malignancy. In the present study, we noticed the different levels of peripheral lymphocytes (CD8 + T, CD8 + CD28 + T, and Treg cells) between those benign and malignant patients.

Regarding the impact of surgery on immune status, a published study showed that removal of primary tumors could reverse tumor-induced immunosuppression despite the presence of metastatic disease [19]. In our study, it was interesting to note that the level of CD8^+^CD28^+^ T cells decreased while B cells increased within 1–2 weeks post-operatively. Xu and colleagues measured pre- and post-operative (4–6 weeks) circulating lymphocyte profile in pancreatic cancer [11]. They found that the post-operative level of CD19^+^ B cells was lower than preoperative, whereas CD8^+^CD28^+^ T cells the opposite [11]. Another study consecutively measured the lymphocyte subsets following open pulmonary lobectomy for lung cancer: before and five, 30 and 60 days after operation [20]. It showed reductions of lymphocytes on post-operative day five, while complete recovery of the preoperative leukocyte setting was documented at 30 and 60 days [20].

The study has several limitations. Firstly, we only measured the lymphocyte subsets in peripheral blood. We planned to evaluate both the circulating and infiltrating lymphocytes in ovarian cancer patients in our future study. Secondly, the sample size is still not large enough. We only included 14 cases of borderline ovarian tumor, which might partly explain the insignificant differences between patients with ovarian cancer and borderline tumor. Thirdly, the survival outcome was not analyzed due to insufficient follow-up. We will update the survival information and investigate the possible prognostic implication of lymphocyte variables. Lastly, one-time snapshot of lymphocyte values could not reflect the kinetics. To better understand the alterations after surgery, consecutive blood samples should be collected.

## Conclusion

We demonstrated the alterations of circulating lymphocyte profile in patients with ovarian cancer. Decreased proportion of CD19^+^ B cells in peripheral blood might be associated with disease aggressiveness, which warrants further assessment.

## Supplementary Information

**Additional file 1:** **Supplementary Figures.** The logic gating and analyzing figures for flow cytometry.

**Additional file 2:** **Supplementary Table 1.** The fluorochrome-conjugated antibodies for flow cytometry.

## Data Availability

The dataset supporting the conclusions of this article is available upon request. Please contact Prof. Huijuan Yang (huijuanyang@hotmail.com).

## Abbreviations

Tregs: Regulatory T cells
NK: Natural Killer
FIGO: The International Federation of Gynecology and Obstetrics
CA: Cancer antigen
CRS: Cytoreductive surgery
