# Supplementary material for: Peripheral lymphocyte populations in ovarian cancer patients and correlations with clinicopathological features
Source: J Ovarian Res. 2022 Apr 11;15:43. doi: 10.1186/s13048-022-00977-3 (PMC8996636; doi:10.1186/s13048-022-00977-3)
Supplement: Supplementary file 1 — Additional file 1: Supplementary Figures. The logic gating and analyzing figures for flow cytometry. [file 13048_2022_977_MOESM1_ESM.pptx]

## Slide 1
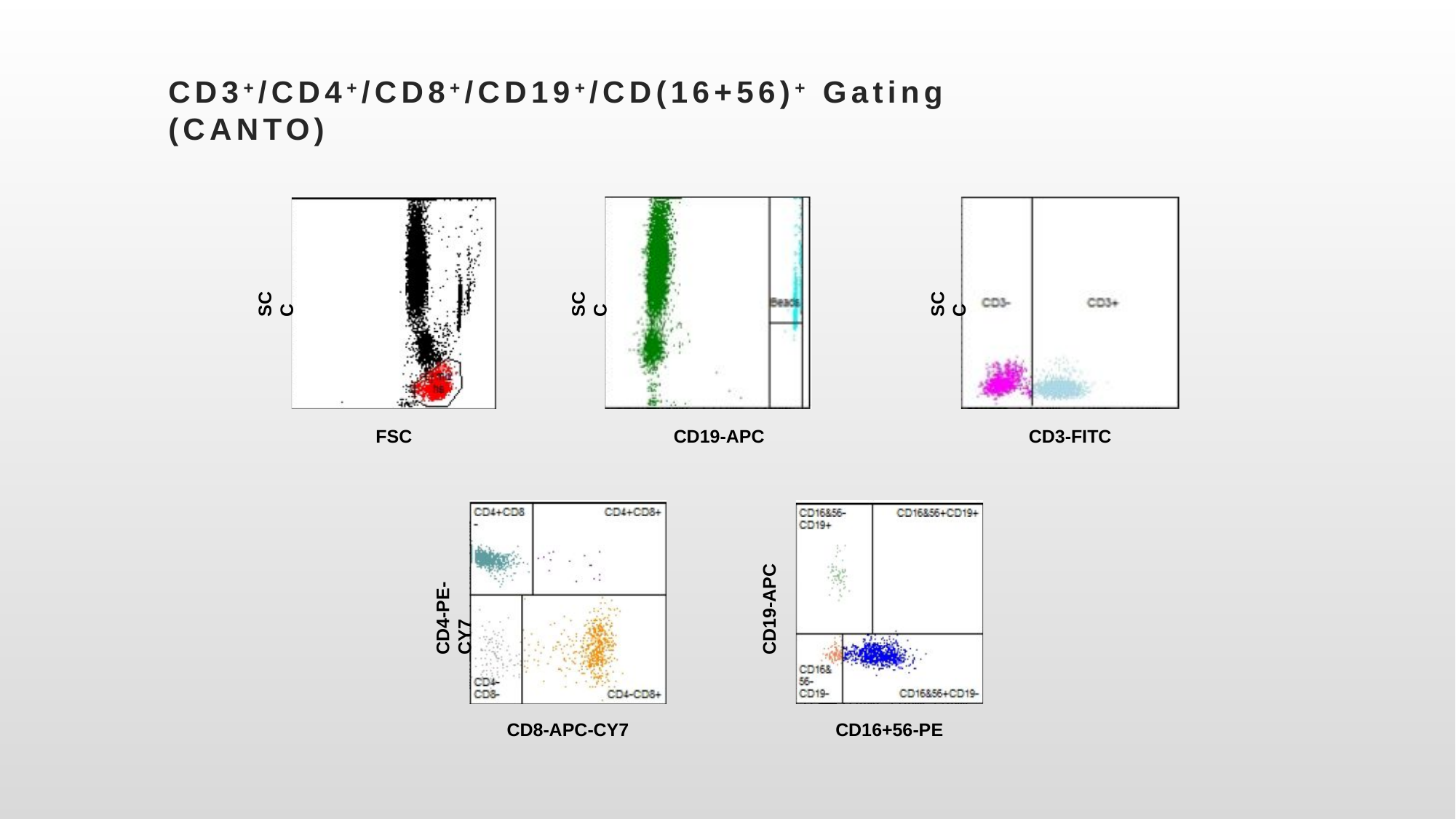

# CD3+/CD4+/CD8+/CD19+/CD(16+56)+ Gating (CANTO)
SCC
CD3-FITC
SCC
FSC
SCC
CD19-APC
CD4-PE-CY7
CD8-APC-CY7
CD16+56-PE
CD19-APC

## Slide 2
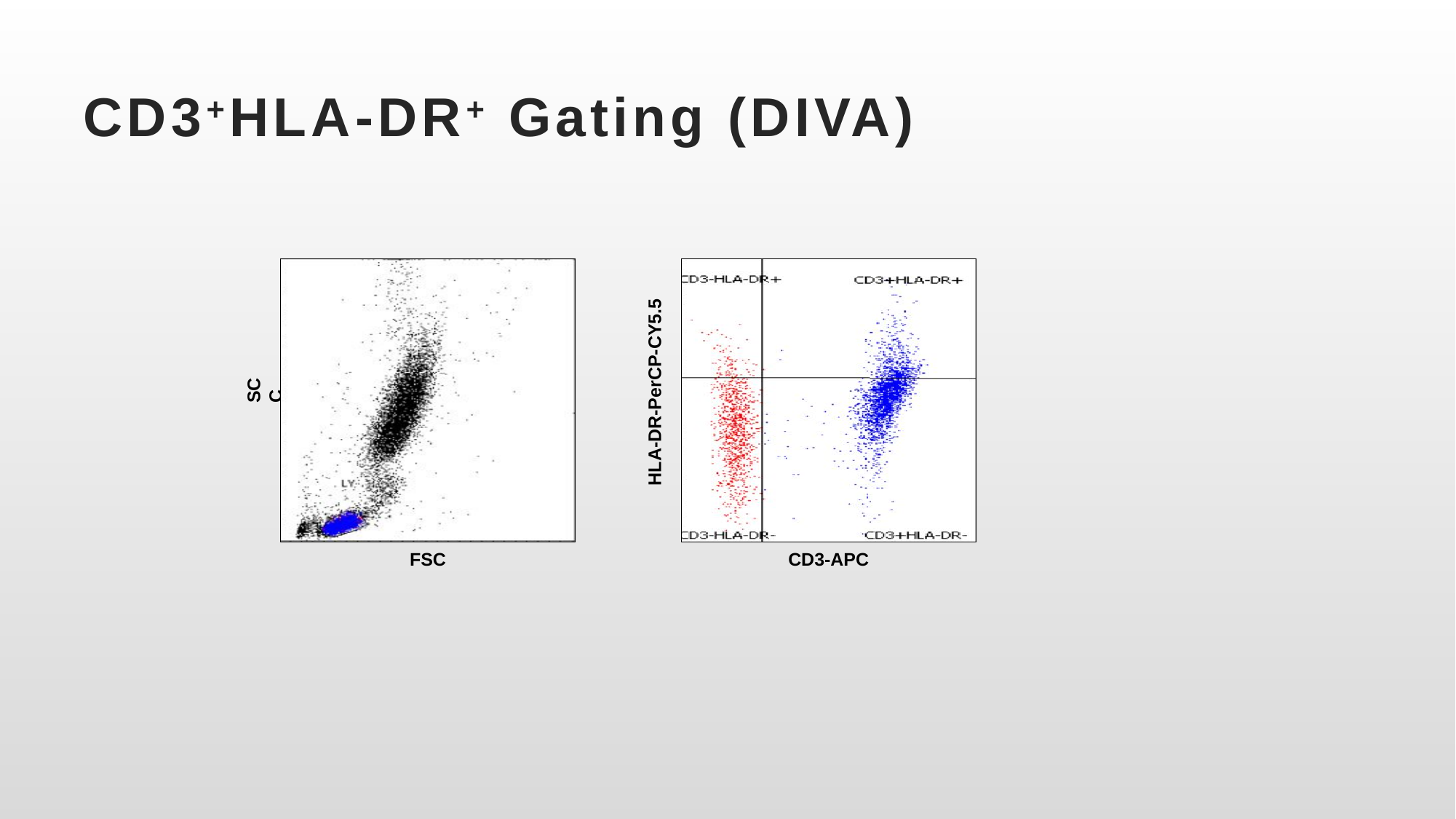

# CD3+HLA-DR+ Gating (DIVA)
SCC
FSC
HLA-DR-PerCP-CY5.5
CD3-APC

## Slide 3
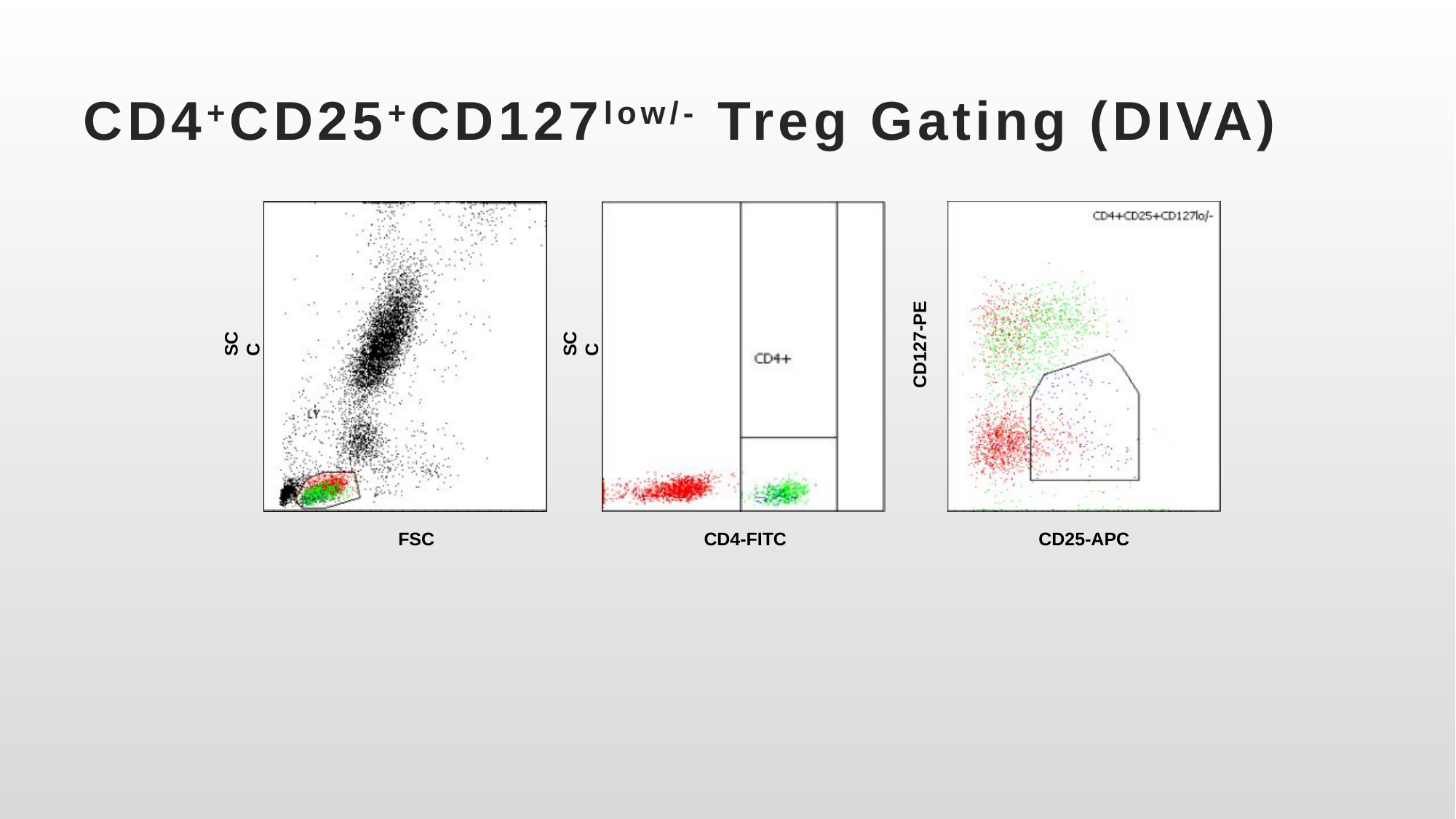

# CD4+CD25+CD127low/- Treg Gating (DIVA)
SCC
FSC
CD127-PE
CD4-FITC
CD25-APC
SCC

## Slide 4
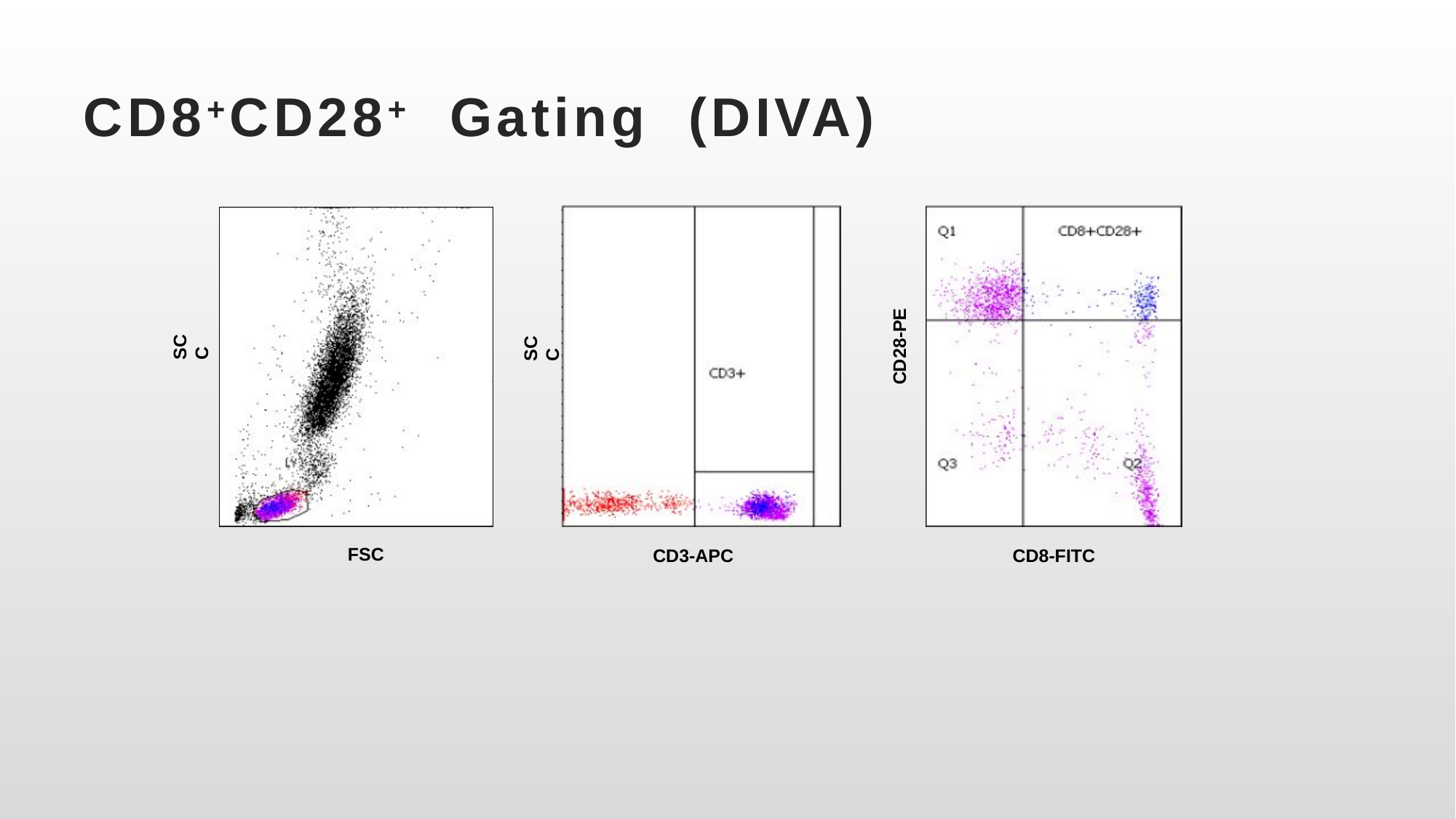

# CD8+CD28+ Gating (DIVA)
CD28-PE
CD3-APC
CD8-FITC
SCC
SCC
FSC

## Slide 5
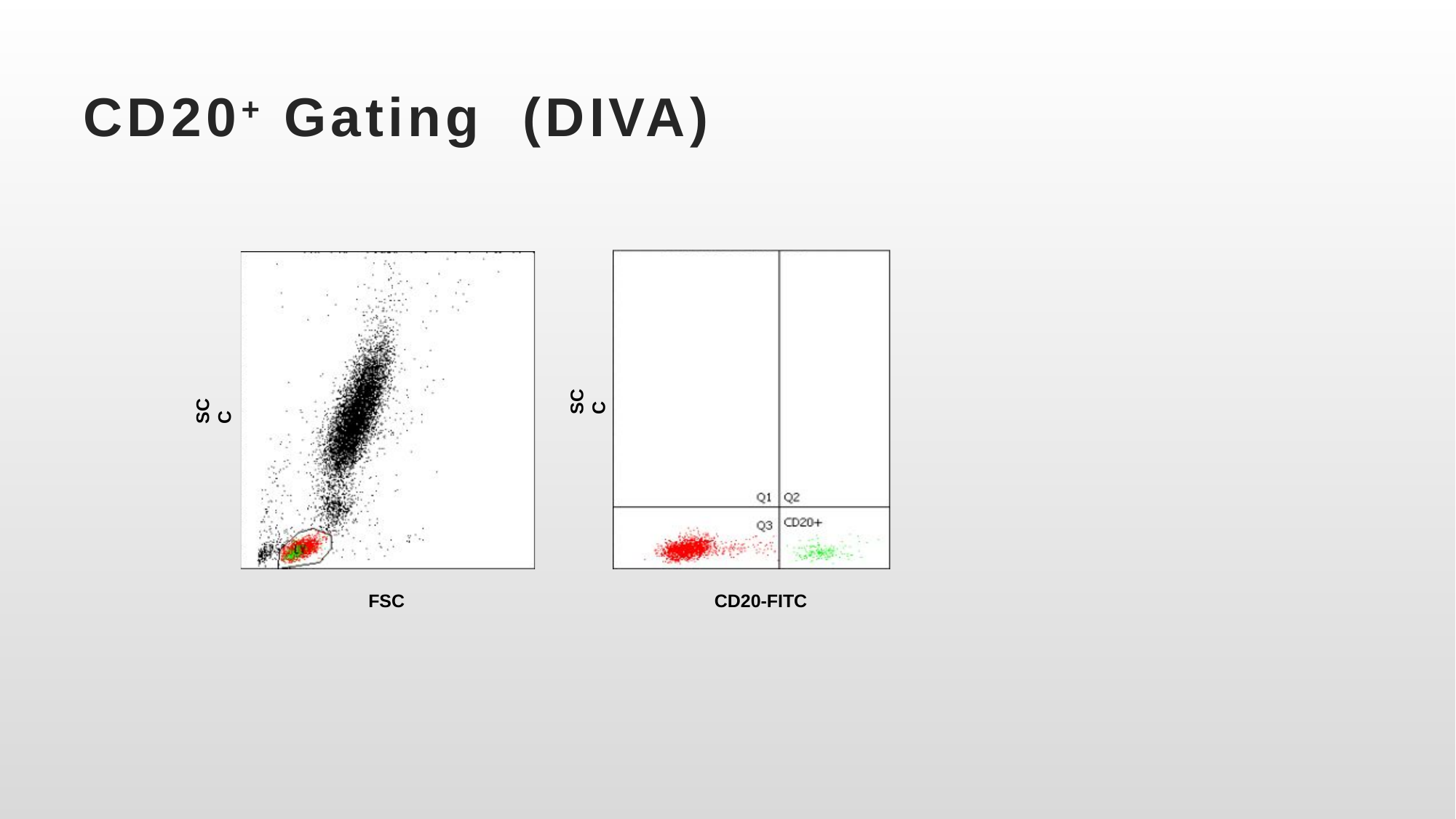

# CD20+ Gating (DIVA)
SCC
CD20-FITC
SCC
FSC
