# Supplementary material for: Peripheral lymphocyte populations in ovarian cancer patients and correlations with clinicopathological features
Source: J Ovarian Res. 2022 Apr 11;15:43. doi: 10.1186/s13048-022-00977-3 (PMC8996636; doi:10.1186/s13048-022-00977-3)
Supplement: Supplementary file 2 — Additional file 2: Supplementary Table 1. The fluorochrome-conjugated antibodies for flow cytometry. [file 13048_2022_977_MOESM2_ESM.docx]

| Supplementary Table 1. The fluorochrome-conjugated antibodies for flow cytometry. | | |
| --- | --- | --- |
| Surface marker | Fluorochrome | Manufacturer |
| CD3 | APC | BD |
| CD4 | FITC | Beckman Coulter（BC） |
| CD8/CD28 | FITC/PE（cocktail） | BD |
| CD20 | FITC | BC |
| CD25 | APC | BD |
| CD127 | PE | BC |
| HLA-DR | PerCP-Cy5.5 | eBioscience |
| CD45/CD3/CD4/CD8/CD19/CD(16+56) | PerCP-Cy5.5/FITC/PE-CY7/APC-CY7/APC/PE（cocktail） | BD |
